# Notes on Health Resorts

**Published:** 1899-10-07

**Authors:** 


### NOTES ON HEALTH RESORTS.

### DROITWICH.

The once old-fashioned market town of Droitwich has made great strides of late years, owing to the growing celebrity of the brine baths for the cure or relief of sufferers from rheumatism, gout, sciatica, lumbago, and similar obstinate complaints. But the town is largely indebted for its prosperity and increasing popularity to Mr. John Corbett, who has been unsparing in energy and liberality for the welfare of the town in which he is a resident and large landowner.

The brine springs of Droitwich were discovered, and it is believed were used as curative agents in the time of the Romans. They have been resorted to for hundreds of years for the cure or relief of rheumatism, but of late years they have become more renowned in consequence of the wide recognition of their efficacy by the medical faculty and the increased and vastly-improved accommodation for patients and visitors which has been provided by those responsible for their management.

Among the various baths and waters which have for long centuries been celebrated for their efficacy in the treatment of disease, those whose principal constituent is chloride of sodium (common salt) have always taken a high position. What happens when the body is bathed in saline waters has been much disputed. It has been broadly asserted by some, indeed, that hardly any absorption of water takes place through the skin in bathing; and, so far as water pure and simple is concerned, that may be true. But there are certain facts which are made manifest in our daily experience which go to prove that substances dissolved in water can penetrate the skin, and can be received into the circulating blood stream. One experience alone, viz., the absorption of carbolic acid, is quite sufficient to prove the point.

Large dressings of carbolised gauze are not employed now as they used to be, but when they were in vogue, and when it was no uncommon thing to see a couple of square feet of skin enclosed in a carbolised dressing, carbolic acid absorption was frequently met with; so that, although it may be perfectly true that water is not absorbed by the skin, and indeed that a man is lighter after a bath than before one, it is none the less true that some interchange takes place between the saline and other constituents of the blood and those of the bath. It is clear, then, that the sort of water bathed in is not a matter of indifference. Nor must it be imagined that the influence of a bath ends as soon as the patient has been rubbed down, for when the bath has been a saline one, and the water has soaked thoroughly into the epidermis, the body surface remains —if one may so say—clothed in salt for a considerable time afterwards. Hence it is that those mineral waters which are included under the heading of muriated or common salt waters are found to be of great medicinal efficacy, even when only used externally. Their internal use, however, is by no means to be overlooked. Taken as a class, it may be said that they exercise "a gently stimulating effect on the gastric and intestinal mucous membrane, increasing peristalsis, and rendering the contents of the bowels more fluid" (Weber). Moreover, in some cases the mere presence of the saline appears to aid the activity of other ingredients which either naturally exist in the water or have been specially added, such as iron or purgative salts. In addition to this, it is believed by some that muriated waters possess a power of facilitating the digestion of albuminous materials, and so aiding in the general nutrition of the body, and, whether this be so or not, there can no doubt that by their diuretic and laxative effects, and the consequent relief to abdominal engorgement, they aid digestion, which is the first step to improved nutrition, and distinctly relieve some cases of dyspepsia. Used externally, the stronger saline or brine baths are employed in various cachectic conditions requiring stimulating treatment, such as scrofula rickets; in catarrhal conditions, whether of the digestive or respiratory system; in emphysema; in many conditions of cardiac weakness, especially when, as at Nauheim, the water is impregnated with a considerable quantity of free carbonic acid, although it must be said that at Nauheim the saline constituents are hardly in such quantity as to warrant the use of the term "brine" bath; lastly, and more especially in the treatment of rheumatoid arthritis, and various gouty and rheumatic affections. There is evidently, then, a large field for the employment of the muriated or common salt baths.

Now it so happens that we possess in England the very type and model of a brine bath. In fact, we possess several, but *facile princeps* among the salt baths of the world stands Droitwich. To give an idea of the saline strength of the Droitwich water it may be mentioned that so far as salt is concerned it is between ten and twelve times as strong as sea water. Indeed, so great is its density that people float about in the baths like cork. They find that they cannot sink, and it is a curious sight to see people floating about on their backs smoking and reading. Even the industry of the ladies need receive no check, for they have been seen doing fancy work while lying as they may do on rather than in the water.

It cannot be said that Droitwich is a very lively place, or capable of comparison, from a watering-place point of view, with some of its foreign competitors. But as a brine bath it is beyond all question far ahead of them, and even in the surroundings which are so necessary for the comfort of its guests, no mean adjuncts, be it said, even to a place which offers the boon of health as a reward to those who make a pilgrimage to its waters, Droitwich has of late made great strides. It has good hotels, golf links, tennis courts, a fine park, with a band in the season, and capital roads for cycling or driving, and, moreover, it is situated in the midst of a very pretty country. With amusements pure and simple, however, it certainly is not well provided, and we fear that those who care neither for billiards nor whist will find the evenings dull; which is a pity, for the rheumatic and gouty patients who form so large a proportion of its clientèle are those who, of all people in the world, most require their pleasures to be brought within their reach. The Droitwich baths can be used at any temperature which is required, the warming being effected by forcing steam through the brine until the desired heat is attained. The usual temperature employed is 90 deg. F. in summer, and 84 deg. in winter. The brine is applied either as "soak," or "needle," or swimming baths, or in the form of a douche, while vapour baths are also given. Massage is also largely used, and there are three fine swimming baths for ladies and gentlemen. This is a matter of some importance, for among the evil consequences of "crippling" affections, whether gouty or rheumatic, is the lack of exercise which such maladies entail. But many of these patients who can only hobble slowly and painfully on foot are practically free from such restraint, and are able to take what really may be quite active exercise, by aid of their unaffected limbs, when swimming in a water so buoyant as the Droitwich brine, so buoyant that they are free from all trouble in regard to the elementary process of keeping afloat. It is more especially in convalescence that the daily swim becomes so useful a means to health, for after all it must be remembered that, in whatever way gout and rheumatism and their congeners may have originated, they are often kept up, and even aggravated by that "torpidity" of liver and other organs which is entailed by prolonged want of exercise, and a complete cure is rendered much more difficult unless a proper amount of muscular exertion is indulged in.

The bathing establishment, comprising the "St. Andrew's" and "Royal" Brine Baths, is large, and the administration efficient. The attendants are experienced and skilled in massage, and every comfort and convenience is provided for the patient. The St. Andrew's Baths, the property of Mr. J. Corbett, have lately been enlarged, and the latest improvements have been carried out.

There is ample accommodation for visitors in private apartments, boarding homes, and hotels. The first-class hotels are the Worcestershire, the Raven, and the Park. The former is a spacious, well-appointed hotel, with good public rooms, and airy bedrooms opening into wide corridors, extending the extreme length of building. It is most conveniently situated, the baths being directly opposite, and the park and golf links within a few minutes' walk.

The length of the Droitwich course of treatment has necessarily to be regulated according to the severity of the disease. The popular course is what is spoken of as the 21 days' cure, but in mild cases and early stages a few baths, with the accompanying massage treatment, have sometimes sufficed to remove the symptoms. It must, however, be stated that in severe and long standing cases the treatment may have to extend over a period of many weeks before any appreciable relief is gained. Nor is it only in cases of rheumatism that the baths are used, for it is found that in persons who are jaded and run down, though otherwise in good health, the stimulating effects of the baths are a capital restorative, while the results are most satisfactory on convalescents, especially those recovering from influenza.

